# Supplementary material for: ProteinShader: illustrative rendering of macromolecules
Source: BMC Struct Biol. 2009 Mar 30;9:19. doi: 10.1186/1472-6807-9-19 (PMC2672931; doi:10.1186/1472-6807-9-19)
Supplement: Additional file 1 — ProteinShader program without source code. This compressed file contains the complete ProteinShader program including associated libraries, but no source code. A README.txt file gives an overview of the ProteinShader distribution, and the index.html file in the help subdirectory has directions on getting started with the program as well as a set of tutorials. [file 1472-6807-9-19-S1.zip › ProteinShader-beta-0_9_4-binary/help/api/org/proteinshader/gui/Renderer.html]

Renderer (ProteinShader API)


|  |  |  |
| --- | --- | --- |
| **Overview** \| **Package** \| **Class** \| **Use** \| **Tree** \| **Deprecated** \| **Index** \| **Help** | |  |
| **PREV CLASS**   NEXT CLASS | **FRAMES**    **NO FRAMES**     **All Classes** |  |
| SUMMARY: NESTED \| FIELD \| CONSTR \| METHOD | DETAIL: FIELD \| CONSTR \| METHOD |  |


---


## org.proteinshader.gui Class Renderer

```
java.lang.Object
  org.proteinshader.gui.Renderer
```

**All Implemented Interfaces:**: EventListener, GLEventListener

---

``` public class Renderer extends Object implements GLEventListener ```

Knows how to render a protein image onto a GLCanvas.

---

| **Field Summary** | |
| --- | --- |
| `static float[]` | `BACKGROUND`             The background color for the canvas is white (1, 1, 1, 0). |


**Constructor Summary**

`Renderer(Mediator mediator)`
  
          Constructs a Renderer.


| **Method Summary** | |
| --- | --- |
| `void` | `blendObjectEdges(boolean b)`             Turns on or off the ability of the halftoning shaders to soften aliasing (jagged edges) by rendering translucent black silhouettes of tube and ribbon segments (and using jitter) before calling on the halftoning shaders. |
| `void` | `cacheGeometricObject(GeometricListInfo info)`             Stores a request to cache a new geomtric object so that the request can be processed the next time display() is called. |
| `void` | `cacheTextLabels(List<String> textLabels)`             Informs the Renderer that new textLabels should be cached because a new Structure has been loaded. |
| `void` | `display(GLAutoDrawable canvas)`             Draws the currently selected Model on the canvas. |
| `void` | `displayChanged(GLAutoDrawable canvas, boolean modeChanged, boolean deviceChanged)`             Called on if the display mode or display device is changed while the program is running. |
| `StructureToGraphics` | `getGraphics()`             Returns a reference to the StructureToGraphics object used by the Renderer. |
| `String` | `getGraphicsCardInfo()`             Returns information on the graphics card that was gathered when the init() method was called. |
| `double` | `getScale()`             Returns the scale factor for the image. |
| `void` | `init(GLAutoDrawable canvas)`             This method is called by the canvas immediately after the OpenGL state is initialized. |
| `boolean` | `isAnimating()`             Returns true if an animation (model rotation) is in progress. |
| `void` | `jitterScene(JitterEnum jitter)`             Draws the scene multiple times to an accumulation buffer while jittering the xy-coordinates a fraction of a pixel. |
| `void` | `moveCameraXY(int x, int y)`             Moves the camera in the x and y directions. |
| `void` | `moveCameraZ(int z)`             Moves the camera in the z-direction. |
| `void` | `printAngstroms(boolean b)`             Setting print angstroms to true will cause object to camera distances to be printed to standard out for testing and debugging purposes (and will also cause tiling numbers to be printed if automatic tiling is in use). |
| `void` | `reshape(GLAutoDrawable canvas, int x, int y, int width, int height)`             Called on when the GLAutoDrawable object has changed its size so that the perspective and viewport can be recalculated. |
| `void` | `rotateAboutXY(double deltaX, double deltaY)`             Rotates the model about the x-axis and y-axis. |
| `void` | `rotateAboutZ(double deltaZ)`             Rotates the model about the z-axis. |
| `void` | `setBackgroundColor(float red, float green, float blue)`             Sets the background color for the canvas. |
| `void` | `setImageOrientation(OrientationEnum orientation)`             Resets the orientation of the image by resetting the angle of rotation about the x-axis and the angle of rotation about the y-axis. |
| `void` | `setNewStructureFlag(boolean flag)`             Informs the Renderer if a new Structure has been loaded so that the camera distance can be reset before the image is shown. |
| `void` | `setScale(double scale)`             Sets the scale factor for the image. |
| `void` | `setVisibleDrawables(Model model, StyleEnum style)`             An array of opaque Segments, and array of opaque Atoms and Bonds, and an array of translucent Drawable objects will be obtained from the Model. |
| `void` | `showAminoAcids(boolean b)`             Controls whether the Renderer will display AminoAcids. |
| `void` | `showHeterogens(boolean b)`             Controls whether the Renderer will display Heterogens. |
| `void` | `showWaters(boolean b)`             Controls whether the Renderer will display Waters. |
| `void` | `startAnimation(double xAxisSpeed, double yAxisSpeed, GLCanvas canvas)`             Uses an Animator object to rotate the protein image about its x-axis and/or y-axis. |
| `void` | `stopAnimation()`             Stops the rotation animation that is started with startAnimation(). |
| `void` | `takeScreenShot(ImageFormatEnum format, File file)`             Sets up a screen shot so that the next call to display() will cause the current image on the canvas to be saved to a JPEG, PNG or GIF file. |
| `void` | `toggleBlendObjectEdges()`             Toggles the boolean value that is set with the blendObjectsEdges() method. |
| `void` | `togglePrintAngstroms()`             Toggles the memory of whether object to camera distances in angstroms (and tiling numbers) should be printed. |

**Methods inherited from class java.lang.Object**

`clone, equals, finalize, getClass, hashCode, notify, notifyAll, toString, wait, wait, wait`

**Field Detail**

### BACKGROUND

```
public static final float[] BACKGROUND
```

:   The background color for the canvas is white (1, 1, 1, 0).


**Constructor Detail**

### Renderer

```
public Renderer(Mediator mediator)
```

:   Constructs a Renderer.

    **Parameters:**: `mediator` - the central Mediator that allows communication with the GUI classes.


**Method Detail**

### init

```
public void init(GLAutoDrawable canvas)
```

:   This method is called by the canvas immediately after the OpenGL
    state is initialized.

    :   **Specified by:**: `init` in interface `GLEventListener`
    :   **Parameters:**: `canvas` - the canvas to draw on.

---


### display

```
public void display(GLAutoDrawable canvas)
```

:   Draws the currently selected Model on the canvas.

    :   **Specified by:**: `display` in interface `GLEventListener`
    :   **Parameters:**: `canvas` - the canvas to draw on.

---


### reshape

```
public void reshape(GLAutoDrawable canvas,
                    int x,
                    int y,
                    int width,
                    int height)
```

:   Called on when the GLAutoDrawable object has changed its size so
    that the perspective and viewport can be recalculated.

    :   **Specified by:**: `reshape` in interface `GLEventListener`
    :   **Parameters:**: `canvas` - the canvas to draw on.: `x` - the x-coordinate of the canvas.: `y` - the y-coordinate of the canvas.: `width` - the width of the canvas.: `height` - the height of the canvas.

---


### displayChanged

```
public void displayChanged(GLAutoDrawable canvas,
                           boolean modeChanged,
                           boolean deviceChanged)
```

:   Called on if the display mode or display device is changed while
    the program is running.

---


### getGraphics

```
public StructureToGraphics getGraphics()
```

:   Returns a reference to the StructureToGraphics object used by the
    Renderer. The address of this graphics object will be needed by
    the Mediator so that controls on the GUI will be able to tell the
    Mediator to cache various OpenGL display lists that are used to
    speed up drawing geometric objects.

    :   **Returns:**: The StructureToGraphics object used by the Renderer.

---


### cacheGeometricObject

```
public void cacheGeometricObject(GeometricListInfo info)
```

:   Stores a request to cache a new geomtric object so that the
    request can be processed the next time display() is called. The
    request is forwarded to the graphics object (StructureToGraphics),
    which will use the info object to generate a new OpenGL display
    list for a geometric object.

    :   **Parameters:**: `info` - describes the geometric object to cache.

---


### moveCameraXY

```
public void moveCameraXY(int x,
                         int y)
```

:   Moves the camera in the x and y directions.

    :   **Parameters:**: `x` - the value to add to the x-coordinate of the camera.: `y` - the value to add to the y-coordinate of the camera.

---


### moveCameraZ

```
public void moveCameraZ(int z)
```

:   Moves the camera in the z-direction.

    :   **Parameters:**: `z` - the value to add to the z-coordinate of the camera.

---


### rotateAboutXY

```
public void rotateAboutXY(double deltaX,
                          double deltaY)
```

:   Rotates the model about the x-axis and y-axis.

    :   **Parameters:**: `deltaX` - the change in angle of rotation about the x-axis.: `deltaY` - the change in angle of rotation about the y-axis.

---


### rotateAboutZ

```
public void rotateAboutZ(double deltaZ)
```

:   Rotates the model about the z-axis.

    :   **Parameters:**: `deltaZ` - the change in angle of rotation.

---


### setBackgroundColor

```
public void setBackgroundColor(float red,
                               float green,
                               float blue)
```

:   Sets the background color for the canvas.

    :   **Parameters:**: `red` - the red component of the background color.: `green` - the green component of the background color.: `blue` - the blue component of the background color.

---


### setImageOrientation

```
public void setImageOrientation(OrientationEnum orientation)
```

:   Resets the orientation of the image by resetting the angle of rotation
    about the x-axis and the angle of rotation about the y-axis. Setting the
    orientation to ORIGINAL is the same as setting it to FRONT, except that
    ORIGINAL will also reset the size of the image.

    :   **Parameters:**: `orientation` - the desired view of the structure (original, front, back, left, right, top, bottom).

---


### setNewStructureFlag

```
public void setNewStructureFlag(boolean flag)
```

:   Informs the Renderer if a new Structure has been loaded so that the camera
    distance can be reset before the image is shown.

    :   **Parameters:**: `flag` - boolean to set true if a new Structure is seen.

---


### cacheTextLabels

```
public void cacheTextLabels(List<String> textLabels)
```

:   Informs the Renderer that new textLabels should be cached because
    a new Structure has been loaded.

---


### setVisibleDrawables

```
public void setVisibleDrawables(Model model,
                                StyleEnum style)
                         throws VisitorException
```

:   An array of opaque Segments, and array of opaque Atoms and Bonds, and an
    array of translucent Drawable objects will be obtained from the Model. The
    style will determine whether Atoms, Bonds, or Segments are included
    in the arrays:

---


### showAminoAcids

```
public void showAminoAcids(boolean b)
```

:   Controls whether the Renderer will display AminoAcids.

    :   **Parameters:**: `b` - boolean value indicating if AminoAcids should be displayed.

---


### showHeterogens

```
public void showHeterogens(boolean b)
```

:   Controls whether the Renderer will display Heterogens.

    :   **Parameters:**: `b` - boolean value indicating if Heterogens should be displayed.

---


### showWaters

```
public void showWaters(boolean b)
```

:   Controls whether the Renderer will display Waters.

    :   **Parameters:**: `b` - boolean value indicating if Waters should be displayed.

---


### startAnimation

```
public void startAnimation(double xAxisSpeed,
                           double yAxisSpeed,
                           GLCanvas canvas)
```

:   Uses an Animator object to rotate the protein image about its x-axis
    and/or y-axis.

    :   **Parameters:**: `xAxisSpeed` - the x-axis speed in degrees per second.: `yAxisSpeed` - the y-axis speed in degrees per second.: `canvas` - the GLCanvas to create an Animator for.

---


### stopAnimation

```
public void stopAnimation()
```

:   Stops the rotation animation that is started with startAnimation().

---


### isAnimating

```
public boolean isAnimating()
```

:   Returns true if an animation (model rotation) is in progress. Otherwise,
    returns false.

    :   **Returns:**: boolean value indicating if an animation is in progresss.

---


### jitterScene

```
public void jitterScene(JitterEnum jitter)
```

:   Draws the scene multiple times to an accumulation buffer while jittering
    the xy-coordinates a fraction of a pixel.

---


### getScale

```
public double getScale()
```

:   Returns the scale factor for the image.

    :   **Returns:**: The scale factor as a double.

---


### setScale

```
public void setScale(double scale)
```

:   Sets the scale factor for the image.

    :   **Parameters:**: `scale` - the scale factor as a double.

---


### takeScreenShot

```
public void takeScreenShot(ImageFormatEnum format,
                           File file)
```

:   Sets up a screen shot so that the next call to display() will cause the
    current image on the canvas to be saved to a JPEG, PNG or GIF file.

    :   **Parameters:**: `format` - the screen shot image format.: `file` - the file to save the screen shot to.

---


### blendObjectEdges

```
public void blendObjectEdges(boolean b)
```

:   Turns on or off the ability of the halftoning shaders to soften aliasing
    (jagged edges) by rendering translucent black silhouettes of tube and
    ribbon segments (and using jitter) before calling on the halftoning shaders.
    This effect may be added to other shaders at a later time.

    :   **Parameters:**: `b` - boolean value to determine if object edges should be smoothed.

---


### toggleBlendObjectEdges

```
public void toggleBlendObjectEdges()
```

:   Toggles the boolean value that is set with the blendObjectsEdges() method.

---


### printAngstroms

```
public void printAngstroms(boolean b)
```

:   Setting print angstroms to true will cause object to camera distances to be
    printed to standard out for testing and debugging purposes (and will also
    cause tiling numbers to be printed if automatic tiling is in use).

    :   **Parameters:**: `b` - true to print to standard out.

---


### togglePrintAngstroms

```
public void togglePrintAngstroms()
```

:   Toggles the memory of whether object to camera distances in angstroms (and
    tiling numbers) should be printed.

---


### getGraphicsCardInfo

```
public String getGraphicsCardInfo()
```

:   Returns information on the graphics card that was gathered when the init()
    method was called.


---


|  |  |  |
| --- | --- | --- |
| **Overview** \| **Package** \| **Class** \| **Use** \| **Tree** \| **Deprecated** \| **Index** \| **Help** | |  |
| **PREV CLASS**   NEXT CLASS | **FRAMES**    **NO FRAMES**     **All Classes** |  |
| SUMMARY: NESTED \| FIELD \| CONSTR \| METHOD | DETAIL: FIELD \| CONSTR \| METHOD |  |


---

# *Copyright © 2007-2008*
